# Targeting DNA-PKcs and telomerase in brain tumour cells

**DOI:** 10.1186/1476-4598-13-232

**Published:** 2014-10-13

**Authors:** Resham Lal Gurung, Hui Kheng Lim, Shriram Venkatesan, Phoebe Su Wen Lee, M Prakash Hande

**Affiliations:** Department of Physiology, Yong Loo Lin School of Medicine, National University of Singapore, 2 Medical Drive, Singapore, 117597 Singapore; Tembusu College, National University of Singapore, Singapore, 138597 Singapore; MRC Genome Damage and Stability Centre, University of Sussex, Falmer, BN19RQ UK

**Keywords:** Telomerase, DNA-PKcs inhibition, Cancer therapy, DNA damage

## Abstract

**Background:**

Patients suffering from brain tumours such as glioblastoma and medulloblastoma have poor prognosis with a median survival of less than a year. Identifying alternative molecular targets would enable us to develop different therapeutic strategies for better management of these tumours.

**Methods:**

Glioblastoma (MO59K and KNS60) and medulloblastoma cells (ONS76) were used in this study. Telomerase inhibitory effects of MST-312, a chemically modified-derivative of epigallocatechin gallate, in the cells were assessed using telomere repeat amplification protocol. Gene expression analysis following MST-312 treatment was done by microarray. Telomere length was measured by telomere restriction fragments analysis. Effects of MST-312 on DNA integrity were evaluated by single cell gel electrophoresis, immunofluorescence assay and cytogenetic analysis. Phosphorylation status of DNA-PKcs was measured with immunoblotting and effects on cell proliferation were monitored with cell titre glow and trypan blue exclusion following dual inhibition.

**Results:**

MST-312 showed strong binding affinity to DNA and displayed reversible telomerase inhibitory effects in brain tumour cells. In addition to the disruption of telomere length maintenance, MST-312 treatment decreased brain tumour cell viability, induced cell cycle arrest and double strand breaks (DSBs). DNA-PKcs activation was observed in telomerase-inhibited cells presumably as a response to DNA damage. Impaired DNA-PKcs in MO59J cells or in MO59K cells treated with DNA-PKcs inhibitor, NU7026, caused a delay in the repair of DSBs. In contrast, MST-312 did not induce DSBs in telomerase negative osteosarcoma cells (U2OS). Combined inhibition of DNA-PKcs and telomerase resulted in an increase in telomere signal-free chromosomal ends in brain tumour cells as well. Interestingly, continual exposure of brain tumour cells to telomerase inhibitor led to population of cells, which displayed resistance to telomerase inhibition-mediated cell arrest. DNA-PKcs ablation in these cells, however, confers higher cell sensitivity to telomerase inhibition, inducing cell death.

**Conclusions:**

Efficient telomerase inhibition was achieved with acute exposure to MST-312 and this resulted in subtle but significant increase in DSBs. Activation of DNA-PKcs might indicate the requirement of NHEJ pathway in the repair telomerase inhibitor induced DNA damage. Therefore, our results suggest a potential strategy in combating brain tumour cells with dual inhibition of telomerase and NHEJ pathway.

## Background

Brain tumours comprise of a wide variety of subtypes of which Glioblastoma multiforme and medulloblastoma are the most common primary malignant brain tumours in adults and children, respectively. Although current clinical data show evidence that molecular targeting has improved the management of brain tumours, prognosis still remains poor [1, 2]. Therefore, the need to discover alternative approach to improve therapeutic response in brain tumour patient remains important.

The two most common hallmarks of tumour cells are their limitless proliferation capacity and sustained massive genome instability [3]. In normal cells, induction of DNA damage either by exogenous or endogenous agents leads to the activation of appropriate DNA damage response (DDR) pathway to ensure immediate repair and to prevent propagation of cells with highly unstable genomes. DNA double-strand breaks (DSB) are the most lethal form of DNA damage and if unrepaired, DSBs severely threaten not only the integrity of the genome but also the survival of the organism [4]. The DDR to DSBs involves activation of homologous recombination (HR) or non-homologous end joining (NHEJ) pathway while the latter is the more dominant DSB repair mechanism compared to HR in mammalian cells [5–7]. NHEJ pathway is facilitated by the DNA-dependent protein kinase (DNA-PK), composed of a catalytic subunit, DNA-PKcs, and the heterodimeric DNA binding regulatory Ku complex, Ku70 and Ku86. The Ku heterodimer binds free DSB ends and recruits DNA-PKcs, which then is activated by the DNA-bound Ku complex. Artemis, the XRCC4/ligase IV complex is also recruited to the complex and serves to catalyse re-ligation of the DNA broken ends. Studies have shown that inhibition of DNA-PKcs sensitise tumour cells to radiation, suggesting its potential as a molecular target in cancer therapy [8].

Besides its role in NHEJ pathway, DNA-PKcs also plays a crucial role in maintaining telomeres [9, 10]. Telomeres are nucleoprotein complexes that function to distinguish the ends of the chromosome from DSBs and to maintain chromosomal integrity [11]. In normal cells, telomere length regulates its replication potential. As cells divide, telomere shortening due to end-replication problem of conventional DNA polymerase, coupled with low or absence of telomerase activity, eventually leads to telomere uncapping, triggering DDR or cell death, if damage is irreparable [12, 13]. In contrast, majority of tumour cells continue to proliferate, despite harbouring relatively shorter telomeres compared to normal tissues due to re-activation of telomerase enzyme [14, 15]. Telomerase complex is made up of the reverse transcriptase catalytic subunit (TERT) and RNA template subunit (TR) and other associated proteins [16]. Inhibition of telomerase via genetic alteration using dominant negative hTERT, mutant hTR template [17, 18] or pharmacological inhibitors slows tumour cell growth by triggering telomere shortening and cell death [19, 20]. Given the high level of telomerase activity in brain tumour progression [21, 22], telomerase provides a molecular target in brain tumour cells.

One major drawback of targeting telomerase is the lag time needed for telomere erosion-mediated cell death leading to possible emergence of adaptive response and resistance mechanisms such as alternative lengthening of telomeres (ALT) mechanism which allows telomere maintenances via HR [23, 24]. Previously, we have shown that inhibition of telomerase and DNA repair protein in mouse embryonic fibroblasts sensitises cells to DNA damaging agents [25]. Here, we provide evidence that efficient telomerase inhibition was achieved using MST-312 in brain tumour cells inducing telomere shortening. Acute telomerase inhibition in brain tumour cells triggered DSBs, cell cycle arrest and subsequent DDR coupled with activation of DNA-PKcs. Inhibition of DNA-PKcs following MST-312 mediated telomere dysfunction leads to increase cell death in brain tumour cells demonstrating the potential therapeutic combinations in enhancing telomerase-mediated therapy.

## Results

### MST-312 reduces telomerase activity and telomere length in brain tumour cells

**Figure 1:**
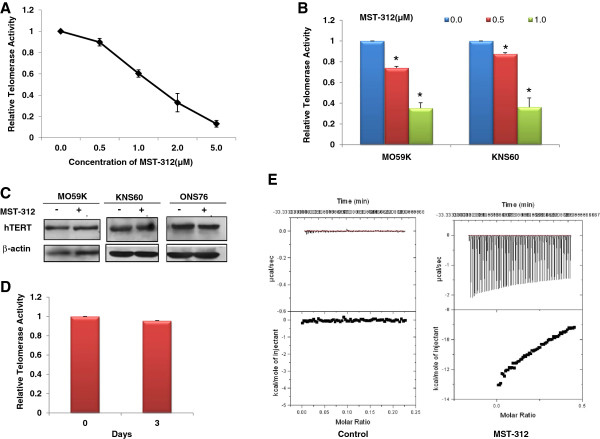
**MST-312 binds to DNA and inhibits telomerase activity in brain cancer cells. (A)** Medulloblastoma cells, ONS76, were treated with indicated doses of MST-312 for 48 hours and examined for telomerase activity by TRAP assay. **(B)** Glioblastoma cells, MO59K and KNS60, were treated with low dose of MST-312 for 48 hours and examined for telomerase activity. **(C)** Brain tumour cells were treated with 1.0 μM MST-312 for 48 hours and the expression of hTERT was determined by western blot. **(D)** MO59K cells treated with 1.0 μM MST-312 for 48 hours were grown in fresh media for 72 hours and telomerase activity was determined. Days refer to the number of recovery days post 48 hours treatment with 1.0 μM MST-312. **(E)** Genomic DNA was extracted from ONS76 cells and binding affinity of MST-312 to DNA determined with ITC assay. ITC assay demonstrated that MST-312 has strong binding affinity to DNA (right panel) demonstrated by the increase in the amount of heat released (bond formation) when normalised with control (left panel). Means and standard errors (SE) from three independent experiments are presented. *Represents statistically significant (p <0.05) in comparison to the respective DMSO treatments.

**Figure 2:**
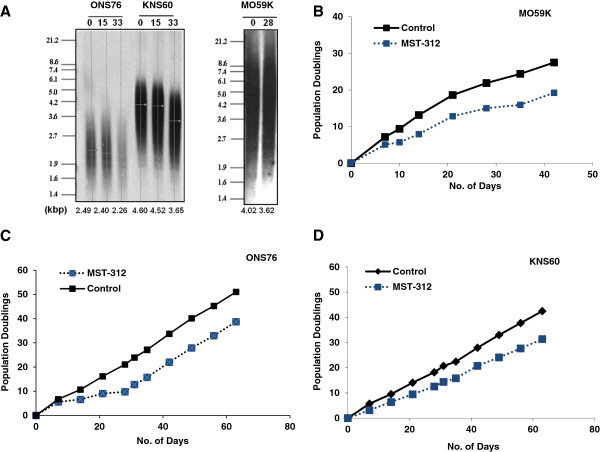
**MST-312 induces telomere shortening and reduces cell proliferation in brain tumour cells. (A)** Total genomic DNA prepared from MO59K, KNS60 and ONS76 cells treated with 0.5 μM MST-312 for indicated number of days was assessed for telomere length using TRF assay. **(B-D)** Cell proliferation capacity in 0.5 μM MST-312 treated brain tumour cells were analysed with trypan blue exclusion assay.

Although, previous studies have shown that MST-312 inhibits telomerase activity in tumour cells, its mode of mechanism is yet to be fully understood in brain tumour cells. We first treated medulloblastoma cells, ONS76 with MST-312 (0–5 μM) for 48 hours and measured the levels of telomerase activity. As shown in Figure 1A, MST-312 inhibited telomerase activity in a dose dependent manner and approximately 40% decrease in telomerase activity was observed at 1.0 μM of MST-312. MST-312 treated glioblastoma cells, MO59K and KNS60, also showed reduction in telomerase activity (Figure 1B). As there was approximately 50% decrease in telomerase activity in all the brain tumour cells tested at 1.0 μM MST-312, subsequent studies were performed using this dose. As telomerase regulation occurs mainly at the level of transcription of its two core subunits, TERT and TR [26] we analysed the expression of TERT and TR following MST-312 treatment. There were no significant changes in *TERT* and *TR* gene expression (data not shown) or TERT protein level following 1.0 μM MST-312 treatment for 48 hours (Figure 1C).Next, we wanted to determine whether telomerase inhibition persists following withdrawal of MST-312 in brain tumour cells. To investigate this, we treated MO59K cells with 1.0 μM MST-312 for 48 hours, after which, cells were grown in MST-312-free media for further 72 hours (recovery period). At the end of 72 hours, telomerase activity in these cells rose back to 95% of basal activity (Figure 1D), indicating that the inhibitory effect of MST-312 is not persistent and is reversible. In addition, we revealed using isothermal calorimetry analysis (ITC) assay that MST-312 has strong binding affinity to DNA (Figure 1E). Taken together, these findings suggest that MST-312 probably acts as a competitive inhibitor to telomerase in brain tumour cells.Telomere length analysis was subsequently carried out in brain tumour cells. Given that cell division is necessary for telomere erosion to occur in the absence or reduced level of telomerase activity, a lower dose of MST-312 was used so that brain tumour cells are still able to proliferate while telomerase activity is being compromised. The brain tumour cells, MO59K, ONS76 and KNS60, were treated with 0.5 μM MST-312. As shown in Figure 2A, a decrease of 0.4 to 0.95 kb in telomere length was observed in brain tumour cells after 4 to 5 weeks of MST-312 treatment. The extent of telomere shortening differed among the various brain tumour cells tested. The smallest reduction (0.23 kb) in telomere length was observed in medulloblastoma cells, ONS76, which had the shortest basal telomere length (Figure 2A). Glioblastoma cells, KNS60, showed the largest decrease (0.95 kb) in telomere length. Next, to examine whether the telomere shortening by the MST compounds was associated with gradual reduction in cell proliferation, we measured the cell count using trypan blue exclusion assay. As shown in Figures 2B-D, there was a gradual reduction in cell proliferation in all the brain tumour cells tested.

### Effects of MST-312 on DNA integrity and cell cycle progression

**Figure 3:**
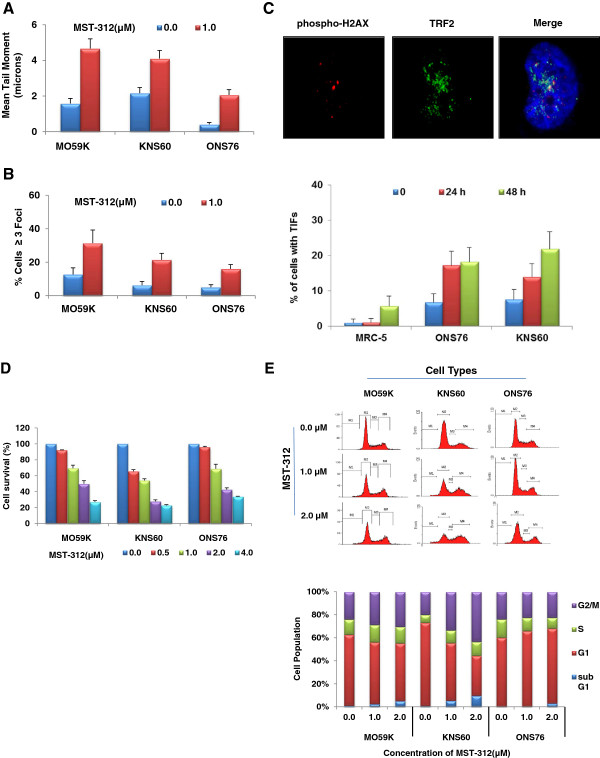
**DNA damage and cell cycle arrest in cells treated with MST-312. (A)** The extent of DNA damage in cells treated with 1.0 μM MST-312 for 48 hours represented by tail moment. A significant increase in DNA damage was observed in all the cell types. **(B)** Extent of double strand breaks was measured using detection of γH2AX foci and Increase in formation of DSBs in all cell types was observed following 48 hours of 1.0 μM MST-312. **(C)** Telomere dysfunction induced foci (TIF) analysis following treatment with 1.0 μM MST-312 for 24 and 48 hours in MRC-5, ONS76 and KNS60 cells. Significant increase in the number of cells with TIFs (yellow foci) was observed following MST-312 treatment in ONS76 and KNS60 cells compared to that in MRC-5 cells. Images were captured using a Zeiss Axioplan Imaging fluorescent microscope with 63 × objective and are processed using Adobe Photoshop CS2 (Adobe Systems Incorporated, USA) for clarity and illustration. **(D)** Decrease in survival of cells was observed in all cells tested following MST-312 concentration as indicated for 48 hours. **(E)** Flow cytometry analysis of cell cycle profiles in MST-312-treated cells. MO59K and KNS60 cells showed significant increase in G2/M and apoptotic cell population. On the contrary, there was significant increase in G1 population in ONS76 cells following MST-312 treatment.

**Figure 4:**
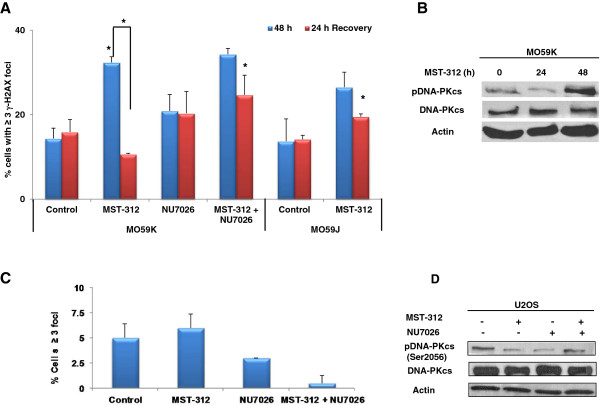
**Role of DNA-PKcs in MST-312-induced double strand breaks in brain tumour cells. (A)** Extent of DSBs was measured as γH2AX foci formation following 1.0 μM MST-312 and/or 10 μM NU7026 treatment. There was significant increase in the distribution of cells positive for γH2AX (damage) in MST-312 treated cells. Following 24 hours recovery (repair) period, the γH2AX level returned to basal level only in DNA-PKcs proficient cells. **(B)** Protein expression studies following (1.0 μM) MST-312 treatment in MO59K cells. Data represent mean ± SE from 3 independent experiments. *P-value <0.05 when compared to untreated controls. **(C-D)** Effects of combined treatment of MST-312 and NU7026 in U2OS cells. **(C)** Level of γH2AX foci positive cells following treatment with 1.0 μM MST-312 and 10 μM NU7026 in telomerase negative U2OS cells. **(D)** DNA-PKcs status following 48 hours treatment with 1.0 μM MST-312 and/or 10 μM NU7026.

Recent studies have shown that short-term telomerase inhibition with MST-312 induces DNA damage as measured by gamma H2AX expression, independent of telomere shortening in primary ependymoma cells [27]. Therefore, we wanted to examine whether the acute telomerase inhibition affects DNA integrity in brain tumour cells. Comet assay was first used to measure the extent of DNA damage following 1.0 μM MST-312 treatment for 48 hours and as summarised in Figure 3A, there was approximately a two-fold increase in the level of DNA damage in all the cells tested. We also determined the phosphorylation of H2AX at serine 139 (γH2AX), a marker for DSBs, and observed significant increase in the number of γH2AX-positive cells (Figure 3B). There was approximately 19%, 15%, 11% increase in γH2AX-positive cells in MST-312 treated MO59K, KNS60 and ONS76 cells as compared to their respective controls. In contrast, telomerase -negative osteosarcoma cells (U2OS) (Figure 4C), and human lung fibroblasts (MRC-5), showed minimal or lesser extent of increase in γH2AX-positive cells (data not shown). Next, telomere-dysfunction induced foci (TIF) analysis was carried following 24 and 48 hours treatment with 1.0 μM MST-312. Telomerase positive brain tumour cells, KNS60 and ONS76 showed greater increase in TIFs as compared to MRC-5 cells (Figure 3C). There was 12% and 14% increase in ONS76 and KNS60 in MST-312 treated cells as compared to 4% increase in MRC-5 cells after 48 hours treatment with MST-312. Given that MST-312 binds to DNA, it is possible that binding of MST-312 to the telomeres delays replication of the cells and activation of DNA damage response pathway.To investigate the consequences of DNA damage following MST-312 treatment, cell cycle progression and cell survival were evaluated. Consistent with dose-dependent decrease in telomerase activity (Figure 1A), there was a dose-dependent decrease in cell viability following MST-312 treatment (Figure 3D). FACS analysis performed in brain tumour cells exposed to MST-312 indicated that inhibition of cell proliferation was related to cell cycle arrest. At 1.0 μM MST-312 treatment for 48 hours, G2/M arrest in glioblastoma cells, MO59K and KNS60, and G1 arrest in medulloblastoma cells, ONS76, were observed. However, there was a significant increase in apoptotic cell death at 2.0 μM MST-312 in both the glioblastoma cells (Figure 3E).

### Effects of MST-312 on mRNA expression in brain tumour cells

DNA damage triggers transcriptional changes in cells in order to maintain genome integrity [28]. Pertinent to this study, genes involved in cell proliferation and signalling, cell death, DNA damage, telomere and telomerase homeostasis were evaluated. As shown in Table 1, a differential expression pattern was observed among the brain tumours cells following MST-312 treatment as compared to their respective controls. For example, in glioblastoma cells KNS60, there was a decrease in *Cyclin B* and *Survivin* gene expression while an increase in both the genes was observed in medulloblastoma cells, ONS76. In addition, the expression of gene coding for interlukin-1 receptor-associated kinase 2 (IRAK2) proteins which activates nuclear factor kappa-B (NF-kB) was up-regulated in glioblastoma cells, KNS60, and down-regulated in medulloblastoma cells, ONS76.

**Table 1:** **List of differentially expressed genes following MST-312 treatment in brain tumour cells**

Gene symbol	KNS60*	ONS76*
**Cell cycle proteins**		
*CCNB1*	**-1.59**	**1.49**
*BIRC5*	**-1.32**	**2.50**
*BIRC6*	**-1.51**	**-1.52**
*ANAPC4*	**-1.40**	1.11
**DNA damage response proteins**		
*ATM*	**-1.58**	**-1.16**
*RAD 50*	**-1.85**	**-1.68**
*BRCA1*	1.02	**1.48**
*PARP1*	**1.11**	1.31
*ERCC5*	**1.27**	-1.04
*BLM*	**-1.59**	1.20
*APTX*	-1.26	**-1.57**
*DAPK3*	1.10	**1.48**
**Telomere and Telomerase**		
*TIN2*	**-1.57**	-1.16
*TERF1*	**1.21**	-1.02
*TERF2*	**1.76**	**-1.48**
*c-MYC*	**-2.75**	**-2.25**
*DKC1*	**-1.19**	**-1.58**
*MRE11A*	**-1.34**	1.00
*RAD50*	**-1.85**	**-1.68**
*RFC1*	**-1.28**	-1.31
*TNKS1BP1*	**1.33**	**-1.82**
*PRPF31*	**-1.39**	1.06
*PRKCA*	**1.40**	**-2.23**
**NF-kB family**		
*NFKBIZ*	**-1.91**	**-2.85**
*NFKBIE*	**1.28**	**1.83**
*NFKB2*	**1.26**	**-1.33**
*NFKBIB*	**1.29**	-1.01
*IRAK2*	**2.25**	**-2.00**
**Tumour necrosis factor**		
*TNFSF10*	-1.17	**-3.80**
*TNFSF12*	**1.30**	-1.01
*MAD1L1*	**1.32**	-1.02
**Transforming growth factor**		
*TGFB2*	**-1.90**	**-1.61**
*TGFBRAP1*	**1.39**	1.18
*TGFB1*	**1.62**	-1.22
*TGFBR3*	**-2.05**	**-2.05**
*SMAD1*	**1.11**	**-1.10**
*SMAD2*	**-1.06**	**-1.10**
*SMAD3*	**1.28**	**-1.51**
*SMAD4*	**1.32**	-1.38
*SMAD5*	**1.04**	-1.34
*SMAD6*	-1.00	**1.49**
*SMAD7*	**-1.96**	**-1.69**
**Mitochondrial function regulators**		
*PGC-1α*	**1.22**	-1.33
*PGC-1β*	1.04	**-1.25**

Functional groupings/clusters of differentially expressed following 48 hours treatment with telomerase inhibitor (1.0 μM MST-312) in glioblastoma cells KNS60 and medulloblastoma cells ONS76. List of selected genes that showed significant fold changes (P <0.05) in at least in one of the tumour cells following MST-312 treatment (*) as compared to their respective controls. Data indicates statistically significant (**bold**) compared with respective untreated controls.

Although we found minimal changes in *TERT* or *TERC* (data not shown) expression in MST-312 treated brain tumour cells, which corroborate with the minimal effects of MST-312 in the TERT protein expression (Figure 1C), we observed down-regulation in proteins reported to have roles in telomerase regulation. For example, a decrease in expression of *c-MYC, TIN2, DKC1,* and *RFC1* genes which are positive regulators of telomerase, was seen in MST-312 treated brain tumour cells. There was also a decrease in expression of Transforming growth factor beta (*TGFB),* a multifunctional cytokine that represses TERT expression via down-regulation on c-myc [29, 30]. Even though we observed down-regulation in expression of *MYC* gene, the level of TERT protein and gene expression was not altered in MST-312 treated brain tumour cells. These findings suggest that the observed anti-proliferative effects following MST-312 treatment in brain tumour cells are via telomerase inhibition rather than TGF-β down-regulation. More importantly, in both the brain tumour cells, KNS60 and ONS76, we observed a consistent decrease in the expression of gene coding for ATM and RAD50 proteins, which are involved in HR pathways.

A recent study carried out in telomerase null mice [31] revealed that peroxisome proliferator-activated receptor gamma, coactivator 1 alpha and beta (*PGC-1α* and *PGC-1β*, also known as *Ppargc1a* and *Ppargc1b*, respectively) were repressed via p53 dependent manner. PGC-1α and PGC-1β are two known regulators of mitochondrial biogenesis and function and repression of either or both decreases mitochondrial biogenesis and function [32]. From our gene expression data, we observed significant changes in PGC1α or PGC1β in brain tumour cells. In medulloblastoma cells ONS76, decreased in expression of PGC1β (p <0.05) was observed following 48 hours MST-312 treatment (Table 1). In contrast, increase in of PGC1α (p < 0.05) was observed in glioblastoma cells KNS60. Given the direct link of p53 activation necessary for repression for p53-induced PGC1α and PGC1β, this differential observation made in our study could be related to different p53 status in ONS76 (wild type p53) and KNS60 (mutant p53) cells [33–35].

### Lack of DNA-PKcs delays repair of MST-312 induced DSBs

Given the importance of DNA-PKcs in NHEJ, we next wanted to investigate the role of DNA-PKcs in repair of DNA damage following telomerase inhibition in brain tumour cells. To evaluate this, we used glioblastoma cells, MO59J, which is deficient in DNA-PKcs activity and MO59K cells, which has wild type DNA-PKcs. We exposed both the cells to 1.0 μM MST-312 for 48 hours and allowed for a 24 hour recovery period to determine if any DSB repair occurred. As shown in Figure 4A, we observed induction of DSBs in both the cell types following 48-hour MST-312 treatment. Interestingly, when DSBs were evaluated in MO59K and MO59J cells 24 hours post MST-312 treatment, MO59J cells failed to repair the DSBs induced by MST-312 as efficiently as in MO59K cells. This finding was further corroborated when inhibition of DNA-PKcs activity in MO59K with NU7026 did not show a similar reduction in DSBs level as observed in MO59K cells (Figure 4A). Then we examined if DNA-PKcs was activated following MST-312 treatment in brain tumour cells. Phosphorylation at Serine 2056 in response to DSBs *in vivo* is important for NHEJ pathway [36]. As shown in Figure 4B, there was a substantial increase in the levels of phospho-DNA-PKcs (Ser-2056) after 48 hour-treatment with 1.0 μM MST-312 in telomerase-positive MO59K cells, unlike in telomerase- negative U2OS cells (Figure 4D). Hence, these findings demonstrate that DNA-PKcs is involved in the repair of DSBs following MST-312 treatment in glioblastoma cells.

### DNA-PKcs inhibition increases telomere dysfunction and cell death in glioblastoma cells

**Figure 5:**
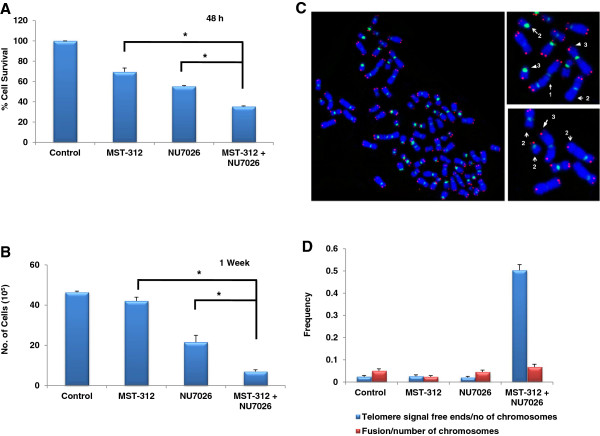
**Inhibition of DNA-PKcs and telomerase increases cell death. (A)** Greatest reduction in cell viability was observed following dual inhibition of telomerase and DNA-PKcs with 1.0 μM MST-312 and 10 μM NU7026 respectively for 48 hours in MO59K cells. **(B)** Synergistic reduction in cell number observed in MST-312 and NU7026 treated cells for one week determined by trypan blue exclusion assay. *P-value <0.05 when compared to untreated controls. **(C)** Partial metaphase spreads from MO59K cells displaying chromosomes stained for telomeres (red) and centromeres (green) with chromosomal fusion doublets (1), absence of telomere signals (2), different telomere intensity between sister chromatids (3). **(D)** Number aberrant telomeres per cell in DNA-PKcs inhibited MO59K cells after 1.0 μM MST-312 treatment for 1 week.

Next, we investigated whether DNA-PKcs inhibition sensitises glioblastoma cells to MST-312 induced cell death. MO59K cells were pre-treated with 10 μM of NU7206 for two hours followed by a 48-hour treatment with 1.0 μM of MST-312. As shown in Figure 5A, inhibition of DNA-PKcs alone led to 45% reduction in cell survival as compared to control cells. This reduction was greater than that in MST-312 treated cells (31%). More importantly, dual inhibition of DNA-PKcs and telomerase led to 65% reduction in terms of cell survival (Figure 5A). To assess further the impact of dual inhibition on glioblastoma cells proliferation, telomerase and DNA-PKcs were inhibited for 1 week in MO59K cells. As shown in Figure 5C, chronic treatment in NU7026 and MST-312 led to synergistic increase in cell death in glioblastoma cells. Cytogenetic analysis was also carried out in these cells to examine the effects of dual inhibition on chromosomal integrity. As shown in Figure 5D, there was minimal increase in chromosomal fusion but approximately 5-fold increase in the frequency of signal-free telomeric ends of chromosomes in cells treated with both NU7026 and MST-312 as compared to untreated control cells.

### Inhibition of DNA-PKcs impair cell proliferation in telomerase inhibited brain tumour cells

**Figure 6:**
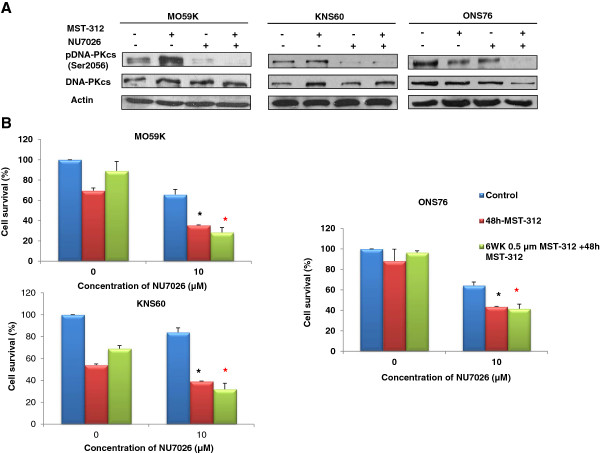
**Activation of DNA-PKcs in MST-312 treated cells and cell survival in brain tumours cells following dual inhibition. (A)** DNA-PKcs activation in MST-312 treated cells determined by western blot analysis following 1.0 μM MST-312 and/or 10 μM NU7026 treatments. Increase in the level of phospho-DNA-PKcs was seen in MO59K and KNS60 cells. On the contrary, minimal changes in the level phospho-DNA-PKcs were observed in ONS76 cells. Representative images from two independent experiments are shown **(B)** Cell survival determined in cells pre-treated with or without low dose MST-312 (0.5 μM) for 6 weeks following which cells were re-plated and treated with MST-312 (1.0 μM) and NU7026 DNA-PKcs inhibition (10.0 μM) for further 48 hours. Combined inhibition of telomerase and DNA-PKcs by MST-312 and NU7026 had the significant reduction in percentage of viable cells for both cells that were pre-treated with a low concentration (0.5 μM) of MST-312 for 6 weeks (6WK) and cells that were not pre-treated. Data represent mean ± SE from 3 independent experiments. *P-value <0.05 when comparing dual inhibited cells to telomerase- inhibited cells for 48 hours. Red star P-value <0.05 when comparing dual inhibited treated cells to telomerase- inhibited cells in pre-MST-312 treated cells.

Our findings in MO59K cells prompted us to investigate the effects of DNA-PKcs inhibition in other telomerase inhibited brain tumour cells. Thus, we first determined the phosphorylation status of DNA-PKcs in KNS60 and ONS76 cells following telomerase inhibition and observed activation of DNA-PKcs only in KNS60 cells (Figure 6A). This may be due to lesser extent of DNA damage in ONS76 cells as compared to KNS60 cells (Figure 3A) leading to cell cycle arrest instead of cell death as observed in KNS60 (Figure 3D-E). Interestingly, there was significant reduction in the level of DNA-PKcs activity in dual inhibited brain tumour cells (Figure 6A) leading to increased growth retardation in MO59K (65%), KNS60 (61%) and ONS76 cells (57%) (Figure 6B).It is suggested that prolonged exposure to telomerase inhibitors may also give rise to population of cells that shows resistance to telomerase inhibition therapy. As shown above, continual treatment with low dose of MST-312 leads to telomere shortening by 28–33 days in brain tumour cells (Figure 2A). Thus, to test the impact of continuous exposure to telomerase inhibitors, brain tumour cells were treated with 0.5 μM of MST-312 for 6 weeks to generate cells with short telomeres. These cells with short telomeres were then exposed to 1.0 μM of MST-312 for 48 hours and cell survival was determined and compared with respective brain tumour cells with long telomeres. As demonstrated in Figure 6B, brain tumour cells pre-treated with 0.5 μM of MST-312 (short telomeres) showed smaller decrease in cell viability as compared to brain tumour cells without MST-312 pre-treatment (long telomeres). Significant reduction in cell viability was only observed in MST-312 pre-treated KNS60 cells as compared to control cells. More importantly, by suppressing DNA-PKcs activity along with telomerase inhibition, the greatest decrease in cell survival was observed in all brain tumour cells (Figure 6B).

## Discussion

Despite harbouring massive genome instability, majority of tumour cells continue to proliferate due to activation of telomerase and subsequent telomere stabilisation [14], In this study, we demonstrated the involvement of DNA-PKcs in DDR pathway following telomerase inhibition in brain tumour cells. Inhibition of telomerase using MST-312 has been shown to induce telomere shortening and impairment of cell proliferation (19). In addition, a study on lung cancer cells revealed that acute telomerase inhibition with MST-312 induced DNA damage [37]. Similarly, we observed DNA damage and cell cycle arrest (Figure 3A-D) following acute telomerase inhibition. Continual exposure of brain tumour cells to telomerase inhibition led to gradual telomere shortening (Figure 2A). However, shortened telomeres were more resistant to telomerase inhibition mediated growth reduction (Figure 6B-D). It has been reported that short telomeres initiate telomere recombination in tumour cells [38] and such recombination can occur in telomerase-positive cells following telomere dysfunction [39]. Although we did not evaluate the mode of telomere maintenance in brain tumour cells after 6 weeks of MST-312 treatment, it is possible that telomere maintenance could also be mediated via ALT pathway as telomere maintenance by telomerase and recombination can co-exist in cells [40].

More importantly, we uncover a link between telomere dysfunction and chemosensitivity specifically towards agents that inhibit DNA repair pathway, providing alternatives to overcome problems associated with long-term exposure to telomerase inhibitors. The sensitizing effects of DNA repair protein are more pronounced in rapidly dividing cells like tumour cells [41] and a recent study has also shown that DNA-PKcs inhibition sensitises breast cancer cells to radiation via telomere capping disruption [42]. In this study, telomerase inhibition in brain tumour cells showed impairment of HR pathway (Table 1) suggesting that DSBs could potentially be repaired by NHEJ. Consistent with this, we observed phosphorylation of DNA-PKcs in brain tumour cells following telomerase inhibition (Figure 6A) and genetic ablation or pharmacological inhibition of DNA-PKcs in glioblastoma cells led to compromised DSBs repair, increased telomere dysfunction and greater cell death (Figures 4 and 5). Dual inhibition of DNA-PKcs and telomerase in glioblastoma, medulloblastoma cells and telomerase-negative osteosarcoma cells, U2OS showed that telomerase-positive brain tumour cells were sensitive to such combinatorial approach as compared to U2OS cells. These data suggest that cells with impaired DNA-PKcs activity appear to enhance a pre-existing telomerase inhibitory effect in brain tumour cells treated with MST-312, leading to accumulation of unrepaired DSBs and reduced cell proliferation (Figures 3 and 6). In addition, MST-312 had relatively minimal effect in non-cancerous, cell lines, MCF10A and MRC-5 (data not shown).

It is important to note that in medulloblastoma cells, ONS76, we did not detect activation of DNA-PKcs following acute telomerase inhibition. In comparison to glioblastoma cells, the relative decrease in telomerase activity was lower in medulloblastoma cells. This could possibly explain the lower level of DSBs induced (Figure 3B). Differential gene expression detected between glioblastoma and medulloblastoma cells clearly suggest some variation in the downstream effects of telomerase inhibition in these brain tumour cells. The genetic background of tumour cells also needs to be assessed as this possibility influences the outcome of treatments. For example, different status of p53 might have influenced the opposite expression profiles of mitochondrial function regulators genes *PGC-1α* and *PGC-1β* in our study.

## Conclusion

Our findings suggest the potential of targeting telomerase and DNA-PKcs as an alternative way for improving tumour radiotherapy. In addition, we showed that such therapeutic approach alleviate problems associated with using telomerase inhibitors alone. Enhanced cytotoxicity observed in both types of brain tumour cells following combined inhibition of telomerase and DNA-PKcs demonstrated that this approach may not be cell-type specific. It is possible that DNA-PKcs inhibition impairs the DNA-PKcs-mediated formation of the end-capping structure at telomeres. This impairment may contribute to telomere dysfunction following telomerase inhibition and compromise the capacity of brain tumour cells to maintain the already complex and unstable genome integrity. We have also seen that the absence of DNA-PKcs (MO59J cells or DNA-PKcs targeted siRNA treated MO59K cells) showed decreased expression of TERT (data not shown), suggesting a possibility of a cross talk between these two proteins in the process. However, further studies using *in vivo* models are warranted and ideal to test the efficacy of such strategy in management of brain tumour cells.

## Methods

### Cell types and cell culture

Human glioblastoma multiforme cells MO59K (CRL-2365) and MO59J (CRL-2366) (American Type Culture Collection, USA) while glioblastoma multiforme cells KNS60, and medulloblastoma cells ONS76 (Institute for Fermentation, IF050357, and IF050355, respectively) were obtained from Dr. Masao Suzuki, National Institute of Radiological Sciences, Chiba, Japan. The cells were cultured in Dulbecco’s Modified Eagles Medium (DMEM) supplemented with 10% heat inactivated foetal bovine serum (Hyclone, USA) and 100 U/ml of penicillin/streptomycin (Gibco, USA). U2OS, ALT-positive osteosarcoma cells (ATCC) were grown in 5A McCoy’s medium, supplemented with 10% foetal bovine serum and L-glutamine (Gibco, USA). All the cells were maintained in a humidified 5% CO_2_ incubator at 37°C. Fresh medium was added after every 2 days and the cell density was kept below 80% confluence.

### Drug treatment

Stock solution of MST-312 (Sigma, USA) [19] and DNA-PKcs inhibitor, NU7026 (Calbiochem, USA) were prepared in dimethyl sulfoxide (DMSO) and suitable working concentrations were made from the stock using complete medium. For short- term experiments, cells were treated with MST-312 and NU7026 for 48 hours. 10 μM of NU7026 was used in this study as previous report has shown that DNA-PKcs activity is completely inhibited at this dose [43].

### Telomere repeat amplification protocol (TRAP)

Brain tumour cells were treated with 0–5 μM MST-312 for 48 hours and telomerase activity detection was performed with the commercially available TRAPeze® XL Telomerase Detection Kit (Chemicon International, USA). All steps were done according to the manufacturer’s instructions. Total protein was extracted from the cell pellet by incubating in CHAPS lysis buffer for 30 minutes on ice. Samples were spun at maximum speed at 4°C for 20 minutes to collect the supernatant. Protein quantification was carried out using Bradford method and 1.5 μg protein was treated with 1 ml/ml RNase inhibitor to eliminate RNase before performing PCR reaction.

### Telomere restriction fragment (TRF) length analysis

Following treatment of brain tumour cells with 0.5 μM MST-312, DNA extraction was performed according to the manufacturers’ protocol using DNeasy Tissue Kit (Qiagen, USA). The telomere restriction fragment length analysis assay was performed using Telo-TAGGG Length Assay Kit (Roche Applied Science, USA). Kodak Gel imaging system and the Kodak imaging software was used to calculate the quantitative measurements of the mean TRF length.

### Alkaline single cell Gel electrophoresis assay

Following treatments with 1.0 μM MST-312, cells were harvested and resuspended in Hank’s Balanced Salt Solution (Sigma) with 10% DMSO and 0.5 M EDTA. The cell suspension was then suspended in 0.7% low melting agarose at 37°C (Conda, Spain), and layered on to comet slides (Trevigen, USA). The cells were then lysed in lysis solution containing 2.5 M NaCl, 100 mM pH 8.0 EDTA, 10 mM Tris–HCl, 1% Triton–X at 4°C for 1 hour. Denaturation was carried out for 40 minutes, in chilled alkaline electrophoresis buffer (pH 13.0-13.7). Electrophoresis was subsequently carried out for 20 minutes. Slides were immersed in neutralization buffer (500 mM Tris–HCl, pH 7.4), dehydrated, dried and stained with SYBR Green dye (Trevigen) and scored with Comet Analysis Software (Metasystems, Germany). The images were captured using Zeiss Axioplan 2 imaging fluorescence microscope (Carl Zeiss, Germany) equipped with triple band filter. One hundred cells were randomly selected and analysed. The extent of DNA damage was expressed as tail moment, which corresponded to the fraction of the DNA in the tail of the comet.

### Immunofluorescence

Briefly, 5 × 10^4^ brain tumour cells were plated in cover slips in six well plates and grown for 48 hours in the presence of 1.0 μM MST-312. Cells were fixed in 4% paraformaldehyde and permeabilise in 0.1% Triton-X-100. Following incubation with anti-phospho-H2AX (Ser139) (Upstate, biotechnology) diluted in PBS with 4% FCS and 0.1% Triton X-100. Cells were washed and incubated with FITC-conjugated anti-mouse secondary antibody (1:500) secondary antibodies at room temperature in the dark for one hour. Subsequent washes were also conducted in the dark. The cover slips were sufficiently dried prior to mounting them onto slides containing Vectashield mounting media with DAPI (Vector laboratories). One hundred cells were randomly selected and screen for the present of foci in each experiments. For the detection of telomere dysfunction induced foci (TIF), cells were fixed as described above with the following changes. Incubation with mouse monoclonal anti- phosphor-H2AX (Millipore)(1:300) was carried out overnight at 4°C, followed by incubation with goat poly anti-TRF2 (Santa Cruz)(1:300) for 1 hour incubation at room temperature. Goat anti-mouse Texas Red (Invitrogen) (1:600) and rabbit anti-goat Fluorescein (Vector Laboratories) were added and incubated for 1 hour at room temperature. Images were captured using a Zeiss Axioplan Imaging fluorescent microscope with 63 × objective and are processed using Adobe Photoshop CS2 (Adobe Systems Incorporated, USA) for clarity and illustration.

### Cell viability assay

A total of 1 × 10^4^ cells per well were plated in transparent 24-well plates (Corning, Costar, USA) and treated with various concentration of MST-312 (0–5 μM) and/or 10 μM NU7026 for 48 hours. Cell viability was measured using CellTiter-Glo® luminescent cell viability assay (Promega, USA) according to the manufacturer’s instructions with some modifications. Briefly, the CellTiter-Glo® reagent was added to cell culture and the mixture were then transferred to white opaque walled 96-well plates (Corning, Costar, USA) for measurement of luminescence using plate reader (Tecan, Switzerland). Cell viability is expressed as percentage of untreated controls.

### Cell proliferation assay

About 1 × 10^4^ cells per well were plated in transparent 12-well plates (Corning, Costar, USA) and 0.5 μM or 1.0 μM of MST-312 and/or 10 μM of NU7026 were added. Fresh drug was added every 72 hours and cell proliferation was determined with trypan blue exclusion assay after indicated number of days.

### Cell cycle analysis

Following 0–2.0 μM MST-312 treatment, cells were harvested, washed in 0.1% BSA: PBS, fixed in 70% ethanol: 1 × PBS, and stained with propidium iodide (Sigma, USA): RNase A (Roche, USA) (2 mg propidium iodide and 2 mg RNaseA/100 mL 0.1% BSA in 1 × PBS). Samples were analysed by flow cytometry (FACSCalibur™, Becton Dickinson, USA) at 488 nm excitation λ and 610 nm emission λ. A total of 10,000 events were captured and data obtained was analysed using Summit 4.3 software. The proportion of cells in different stages of cell cycle is expressed in terms of percentage of total cells analysed.

### Western blot analysis

Total cellular proteins were isolated following a 48 hour-treatment with 1.0 μM of MST-312 and/or 10 μM of NU7026, using RIPA (radio-immunoprecipitation assay) buffer (1% nonidet P-40, 1% sodium deoxycholate, 0.1% SDS, 0.15 M NaCl, 0.01 M sodium phosphate, 2 mM EDTA, 50 mM sodium fluoride, 0.2 mM sodium vanadate and 100 U/ml aprotinin, pH 7.2) from control and treated cells. The whole cell lysate was recovered by centrifugation at 14,000 rpm for 10 minutes. Protein concentration was determined by the bicinchoninic acid method using an assay kit (Pierce Biotechnology, USA) with bovine serum albumin as a standard. Western blot analyses of, DNA-PKcs, p-DNA-PKcs(Ser2056), (phospho DNA-PKcs, Abcam, UK,1:1000) hTERT (Epitomics, USA), and β-actin were performed using specific antibodies from Santa Cruz Biotechnology, USA unless otherwise stated.

### Peptide nucleic acid fluorescence *in situ*hybridisation (PNA-FISH) analysis

For cytogenetic analysis, MO59K cells were treated with 1.0 μM of MST-312 and/or 10 μM of NU7026 for 1 week and cells were arrested at mitosis by treatment with colcemid (0.1 mg/ml). Cells were subsequently incubated with a hypotonic solution of potassium chloride at 37°C for 15 minutes followed by fixation in Carnoy’s fixative. Fluorescence *in situ* hybridisation (FISH) was performed using telomere sequence-specific peptide nucleic acid (PNA) probe labelled with Cy3 as described [44, 45]. Metaphase spreads were captured using Zeiss Axioplan 2 Imaging fluorescence microscope and analysed using ISIS Software (Metasystems, Germany) for telomere-mediated chromosome alterations.

### Gene expression analysis

Following treatment with 1.0 μM of MST-312 for 48 hours, the total RNA was extracted from KNS60 and ONS76 cells using QIAmp RNA Blood Mini Kit (Qiagen, Hilden, Germany). The extracted RNA was quantified using NanoDrop 1000 (Thermo Scientific, USA). RNA integrity was checked using Bio-Analyzer (Agilent Technologies, Inc., USA). Five hundred nanograms of extracted RNA from each sample were used for gene expression study. TotalPrep RNA Amplification Kit (Ambion Inc., TX, USA) was used for cRNA amplification process. The biotinylated amplified RNA thus generated was used for hybridization with HumanRef8 V3.0, Human Whole-Genome Expression BeadChips (Illumina Inc., USA) for 16 hours at 58°C. After the incubation period, the arrays were washed and stained with Streptavidin-Cy3 (GE Healthcare, Bio-Sciences, UK). Illumina Bead Array Reader was used to scan the arrays. The array data thus obtained after scanning was imported and analysed using Partek® Genomics Suite™ (Partek GS) (Partek Incorporated, MO, USA).

### Statistical analysis

Statistical significance in the data sets was assessed by Student’s *t*-test (Microsoft Corporation, USA) and two-way ANOVA using Graphpad Prism. The difference was considered to be statistically significant when p < 0.05.

## Abbreviations

EGCG: Epigallocatechin gallate
DDR: DNA damage response
DSBs: Double-strand breaks
HR: Homologous recombination
NHEJ: Non-homologous end joining
DNA-PK: DNA-dependent protein kinase
TERT: Reverse transcriptase catalytic subunit
ALT: Alternative lengthening of telomeres.
